# Electronic Health Record–Based Algorithm for Monitoring Respiratory Virus–Like Illness

**DOI:** 10.3201/eid3006.230473

**Published:** 2024-06

**Authors:** Noelle M. Cocoros, Karen Eberhardt, Vu-Thuy Nguyen, Catherine M. Brown, Alfred DeMaria, Lawrence C. Madoff, Liisa M. Randall, Michael Klompas

**Affiliations:** Harvard Pilgrim Health Care Institute, Boston, Massachusetts, USA (N.M. Cocoros, V.-T. Nguyen, M. Klompas);; Harvard Medical School, Boston (N.M. Cocoros, M. Klompas);; Commonwealth Informatics, Waltham, Massachusetts, USA (K. Eberhardt);; Massachusetts Department of Public Health, Boston (C.M. Brown, A. DeMaria, L.C. Madoff, L.M. Randall);; Brigham and Women’s Hospital, Boston (M. Klompas)

**Keywords:** respiratory infections, viruses, electronic health records, algorithms, disease surveillance, respiratory virus–like illness, RAVIOLI, influenza-like illness, Massachusetts, United States

## Abstract

Viral respiratory illness surveillance has traditionally focused on single pathogens (e.g., influenza) and required fever to identify influenza-like illness (ILI). We developed an automated system applying both laboratory test and syndrome criteria to electronic health records from 3 practice groups in Massachusetts, USA, to monitor trends in respiratory viral–like illness (RAVIOLI) across multiple pathogens. We identified RAVIOLI syndrome using diagnosis codes associated with respiratory viral testing or positive respiratory viral assays or fever. After retrospectively applying RAVIOLI criteria to electronic health records, we observed annual winter peaks during 2015–2019, predominantly caused by influenza, followed by cyclic peaks corresponding to SARS-CoV-2 surges during 2020–2024, spikes in RSV in mid-2021 and late 2022, and recrudescent influenza in late 2022 and 2023. RAVIOLI rates were higher and fluctuations more pronounced compared with traditional ILI surveillance. RAVIOLI broadens the scope, granularity, sensitivity, and specificity of respiratory viral illness surveillance compared with traditional ILI surveillance.

Respiratory viral illnesses place an enormous burden on human health and the healthcare system (*1*–*3*). Although multiple pathogenic respiratory viruses circulate, often simultaneously, public health has traditionally dedicated most of its attention to monitoring trends in laboratory-confirmed influenza and influenza-like illness (ILI). Illness and death associated with seasonal respiratory syncytial virus (RSV) spikes, the SARS-CoV-2 pandemic, and occasional clusters of infection from other respiratory pathogens, however, illustrate the importance of expanding monitoring to include all respiratory viral–like illness activity. Relying on laboratory testing alone will not accomplish this goal because most persons with respiratory viral illnesses do not seek care, many who do seek care are not tested, and not everyone tested is tested for all respiratory viruses. 

Public health agencies have traditionally relied on syndromic surveillance to monitor conditions for which testing rates are low and variable (*4*). The Centers for Disease Control and Prevention’s outpatient Influenza-like Illness Surveillance Network and emerging systems designed to monitor COVID-19–like illness are prime examples (*5*–*9*). However, syndromic surveillance systems tend to provide little or no information about which particular pathogens are circulating, and most jurisdictions require fever to define ILI, a requirement that increases specificity but lowers sensitivity (fever occurs in fewer than half of persons with laboratory-confirmed influenza) (*10*). Surveillance focusing on single pathogens (e.g., influenza, SARS-CoV-2), viral testing alone, or syndromic definitions alone provides an incomplete picture of respiratory illness activity and can miss critical trends and developments (*11*,*12*). Extending surveillance to include multiple pathogens, using both laboratory testing and syndromes, and decreasing reliance on fever as a gatekeeper symptom are necessary to provide public health agencies and healthcare institutions with the data needed to improve situational awareness for planning, resource use, internal and external communications, and targeted prevention activities. 

To regularly monitor overall respiratory viral illness activity associated with multiple pathogens, we developed an integrated surveillance strategy using a combination of laboratory and syndromic indicators, incorporating logic to identify the relative contributions of different individual pathogens. We describe our data-driven approach to developing a routine, automated respiratory virus-like illness (RAVIOLI) algorithm for syndromic surveillance in Massachusetts using live electronic health record (EHR) data drawn from 3 large practice groups. Our work was performed as public health surveillance and therefore not subject to institutional review board oversight. 

## Methods

We used the Electronic Medical Record Support for Public Health (ESP, https://www.esphealth.org) public health surveillance platform to develop the RAVIOLI algorithm. ESP is open-source software that uses automated daily extracts of EHR data to identify and report conditions of public health interest to health departments (*13*–*17*). ESP maps raw data to common terms and then applies algorithms to identify conditions using diagnosis codes, prescriptions, laboratory tests, and vital signs. In Massachusetts, ESP is used for automated reporting of infectious disease cases to the Massachusetts Department of Public Health, aggregate reporting of chronic diseases, and continuum-of-care assessments (*18*–*21*). 

Three multisite clinical practice groups that use ESP for infectious disease reporting, Atrius Health, Cambridge Health Alliance, and Boston Medical Center, contributed data for our project. Atrius Health (https://www.atriushealth.org) is an ambulatory care group with >30 locations in eastern Massachusetts that provides clinical services for a population of ≈700,000. Cambridge Health Alliance (https://www.challiance.org) is a safety-net system that provides ambulatory and inpatient care to >140,000 patients in communities north of Boston. Boston Medical Center (https://www.bmc.org) is a 514-bed academic medical center and safety-net hospital that provides ambulatory and inpatient care to ≈220,000 persons. We combined data from those 3 sites for this analysis.

We sought to develop an evidence-based set of diagnosis codes to identify respiratory virus–like illnesses and assess whether a subset of those codes might be predictive of specific pathogens. To identify codes associated with respiratory viral illness syndrome, we identified all patients tested for respiratory viruses (Table 1) during October 3, 2015–July 30, 2022. Among patients who tested positive for >1 virus, we identified all International Classification of Diseases, 10th Revision (ICD-10), diagnosis codes recorded within 2 days before or after the specimen collection date. For patients without a recorded specimen collection date, we used the test order date; if that was unavailable, we used the result date. We manually removed ICD-10 codes unrelated to respiratory viral illness (e.g., trauma, cancer, chronic disease management). The list of >7,000 excluded codes is available upon request from the authors. 

**Table 1:** Respiratory pathogens and test types included in RAVIOLI algorithm for monitoring respiratory virus–like illness*

Pathogen	Test types
Adenovirus	NAAT
Non–SARS-CoV-2 coronaviruses: OC43,229E, HKU1, NL63	NAAT
Human metapneumovirus	NAAT
Influenza	NAAT, antigen/rapid, culture
Parainfluenza	NAAT
Respiratory syncytial virus	NAAT, antigen
Rhinovirus/enterovirus	NAAT
SARS-CoV-2	NAAT, antigen/rapid

*Respiratory virus–like illness is defined as a clinical encounter with a positive laboratory test result for a respiratory virus, as shown in this table; 1 of the International Classification of Diseases, 10th Revision, diagnosis codes shown in Table 1; or a measured fever >100°F. NAAT, nucleic acid amplification test.

We calculated the positive predictive value (PPV) for each ICD-10 code associated with positive respiratory virus test results. We also calculated the PPV for measured temperature >100°F within 2 days before or after a positive respiratory virus test. We calculated the PPV for each ICD-10 code and fever as the number of encounters with the diagnosis code within 2 days of a positive test divided by the total number of times the diagnosis code occurred across  all clinical encounters during the study period. We defined a clinical encounter as a patient receiving a relevant diagnosis code, immunization, vital sign measure, laboratory test, or prescription. 

We included in the final algorithm diagnosis codes with a PPV ≥10% for any respiratory virus (all viruses combined) or for a specific individual respiratory virus. We also included encounters with positive respiratory virus tests in the total count of respiratory virus encounters as well as in virus-specific categories of RAVIOLI. We counted each viral encounter only 1 time if the patient had both a positive respiratory virus assay result and >1 suggestive diagnosis code. We classified measured fever alone and diagnosis codes with a PPV of ≥10% for any positive respiratory virus test but <10% for any specific respiratory virus in a category referred to as nonspecific for respiratory viral illness syndrome. In summary, we categorized positive cases within RAVIOLI as virus-specific (e.g., influenza, adenovirus), based on a positive test or a diagnosis code with a PPV ≥10% for the specific virus, or nonspecific, based on fever or a diagnosis code with a PPV ≥10% for any positive test of interest. 

To better understand the underlying data included in the final RAVIOLI algorithm, we examined the proportion of patients in each virus-specific category of the algorithm with a positive laboratory test and the proportion of patients in the nonspecific category with a fever. We generated weekly counts during October 3, 2015–January 13, 2024, for clinical encounters with patients meeting the RAVIOLI algorithm, overall and stratified by the probable etiology when possible. For comparison, we also identified the proportion of patients that met the definition of ILI: fever and a diagnosis code for any influenza-like symptom or diagnosis; fever was identified by either a measured fever >100°F or diagnosis code for fever (Appendix Table 1). 

## Results 

**Figure 1:**
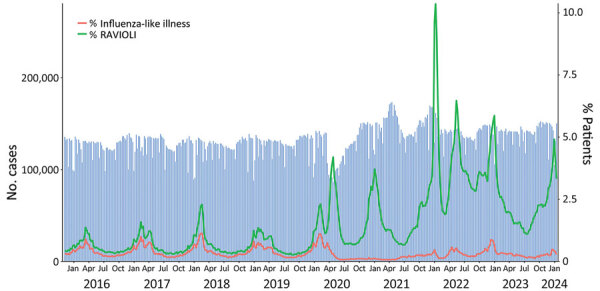
Numbers of patients with a clinical encounter for respiratory virus–like illness and the percentages that met the requirements for influenza-like illness versus those of the RAVIOLI algorithm for monitoring respiratory virus–like illness, by week, Massachusetts, USA, October 2015–January 2024. Patients receiving a diagnosis code, immunization, vital sign measure, laboratory test, or prescription were considered to have a clinical encounter.

Forty-two diagnosis codes (Table 2) and measured fever (>100°F) had a PPV ≥10% for either any positive respiratory virus test (nonspecific) or >1 virus-specific positive test; those diagnosis codes and fever are included in the RAVIOLI algorithm. We recorded weekly counts of patients with clinical encounters and calculated the proportion that met the definition for RAVIOLI overall (diagnosis code, fever, or positive respiratory virus test) and, for comparison, the proportion that met the ILI criteria (Figure 1). The percentage of encounters that met the RAVIOLI algorithm showed clear seasonal trends of annual winter spikes during 2015–2019 followed by periodic increases during spring 2020–early 2024, corresponding to emergence or surges of SARS-CoV-2, RSV, and influenza in Massachusetts. RAVIOLI was identified in a much larger proportion of encounters than ILI after March 2020 and, at times (e.g., fall 2021, August–November 2023), ILI did not detect an increase in respiratory virus illness while RAVIOLI did. 

**Table 2:** ICD-10 diagnosis codes that met the positive predictive value threshold for confirmed respiratory viral illnesses and are included in the RAVIOLI algorithm for monitoring respiratory virus–like illness*

Virus	ICD-10 codes†	Description
Adenovirus	A08.2	Adenoviral enteritis
	B34.0	Adenovirus infection, unspecified
	B97.0	Adenovirus as the cause of diseases classified elsewhere
	J12.0	Adenoviral pneumonia
Non–SARS-CoV-2 coronavirus	B34.2	Coronavirus infection, unspecified
SARS-CoV-2	B34.2	Coronavirus infection, unspecified
	B97.29	Other coronavirus as the cause of diseases classified elsewhere
	J12.82	Pneumonia associated with coronavirus disease 2019
	J12.89	Other viral pneumonia
	J80	Acute respiratory distress syndrome
	R05.1	Acute cough
	R48.1	Agnosia
	U07.1	COVID-19
Human metapneumovirus	B97.81	Human metapneumovirus as the cause of diseases classified elsewhere
	J12.3	Human metapneumovirus pneumonia
	J21.1	Acute bronchiolitis associated with human metapneumovirus
Influenza	J09.X1	Influenza from identified novel influenza A virus with pneumonia
	J09.X2	Influenza associated with identified novel influenza A virus with other respiratory manifestations
	J10.00	Influenza associated with other identified influenza virus with unspecified type of pneumonia
	J10.1	Influenza associated with other identified influenza virus with other respiratory manifestations
	J11.00	Influenza associated with unidentified influenza virus with unspecified type of pneumonia
	J11.1	Influenza associated with unidentified influenza virus with other respiratory manifestations
Parainfluenza	B33.8	Other specified viral diseases
	B34.8	Other viral infections of unspecified site
	J20.4	Acute bronchitis associated with parainfluenza virus
Rhinovirus and enterovirus	B34.0	Adenovirus infection, unspecified
	B34.8	Other viral infections of unspecified site
	B97.10	Unspecified enterovirus as the cause of diseases classified elsewhere
	J20.6	Acute bronchitis associated with rhinovirus
	J45.902	Unspecified asthma with status asthmaticus
Respiratory syncytial virus	B97.4	Respiratory syncytial virus as the cause of diseases classified elsewhere
	J12.1	Respiratory syncytial virus pneumonia
	J20.5	Acute bronchitis associated with respiratory syncytial virus
	J21.0	Acute bronchiolitis associated with respiratory syncytial virus
Any respiratory viral test (nonspecific)	J21.8	Acute bronchiolitis associated with other specified organisms
	R06.03	Acute respiratory distress
	P81.9	Disturbance of temperature regulation of newborn, unspecified
	J12.9	Viral pneumonia, unspecified
	R50.81	Fever manifesting with conditions classified elsewhere
	J96.90	Respiratory failure, unspecified, unspecified whether with hypoxia or hypercapnia
	R05.9	Cough, unspecified
	J96.91	Respiratory failure, unspecified with hypoxia
	J96.92	Respiratory failure, unspecified with hypercapnia
	R57.9	Shock, unspecified

*Respiratory virus-like illness is defined as a clinical encounter with a positive laboratory test result for a respiratory virus listed in Table 1; 1 of the ICD-10 diagnosis codes listed in this table; or a measured fever >100°F. ICD-10, International Classification of Diseases, 10th Revision. †All of the diagnosis codes in the table had a positive predictive value ≥10% PPV for either any positive respiratory virus laboratory test or 1 of the virus-specific positive tests.

**Figure 2:**
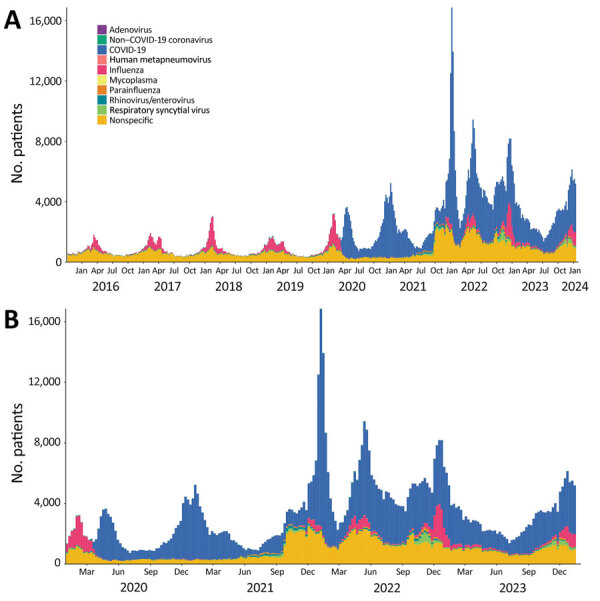
Numbers of patients that met the requirements for the RAVIOLI algorithm for monitoring respiratory virus–like illness, by pathogen category and week, Massachusetts, USA, October 2015–January 2024. A) October 2015–January 2024; B) January 2020–January 2024. Within each virus-specific category are counts of positive test results and diagnosis codes with a positive predictive value (PPV) ≥10% for that specific pathogen. The nonspecific category includes diagnosis codes with a PPV of ≥10% for any positive respiratory viral assay but PPV of <10% for any specific respiratory virus and includes measured fever >100°F.

**Figure 3:**
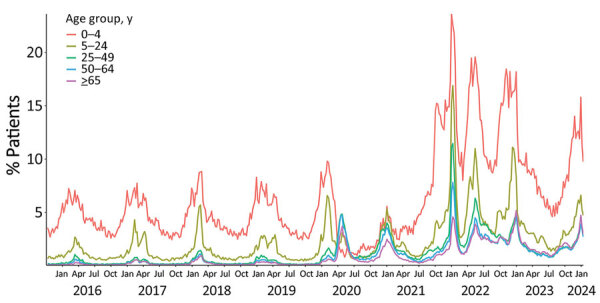
Percentage of patients meeting the RAVIOLI algorithm for monitoring respiratory virus–like illness, by age group, Massachusetts, USA, October 2015–January 2024.

We estimated weekly counts of patients with clinical encounters meeting the RAVIOLI algorithm stratified by encounters with virus-specific or nonspecific encounters without a classified virus. We calculated those data for the full study period, October 2015–January 2024 (Figure 2, panel A), and for January 2020–January 2024 (Figure 2, panel B). Before March 2020, most RAVIOLI encounters came from the influenza or nonspecific categories. SARS-CoV-2 subsequently dominated until fall 2021, when the nonspecific category reemerged, along with influenza and RSV. When we examined trends by patient age groups, the highest proportion of encounters that met the RAVIOLI algorithm were among children 0–4 years of age, followed by young persons 5–24 years of age (Figure 3). 

Data from January 2023–January 2024 show the proportions of patients in the COVID-19, influenza, and RSV categories with a positive laboratory test versus diagnosis code, as well as the proportion in the nonspecific category with fever (Appendix Table 2). The proportion with a positive test varied by virus and time; patients in the COVID-19 category were least likely and those in the RSV category most likely to have a positive laboratory test. Among patients in the nonspecific category, one third or fewer had evidence of fever, and most were identified by a diagnosis code. We also determined the proportion of RAVIOLI patients identified on the basis of >1 positive laboratory test, diagnosis code, or fever during January 2021–January 2024 (Appendix Figure 1); RAVIOLI patients can meet >1 criterion (e.g., have both a positive laboratory test and a diagnosis code). Diagnosis codes were the most common element contributing to identification in most weeks, followed by positive laboratory tests and fever. 

## Discussion 

Respiratory viruses continue to impose a high burden on patients, healthcare providers, and society, and multiple pathogens, including SARS-CoV-2, influenza, RSV, and others, contribute to the burden of respiratory illnesses. Both healthcare providers and public health agencies therefore have an interest in having access to timely and granular data on trends in respiratory viral illnesses and contributing pathogens. We developed an EHR-based algorithm for integrated surveillance of respiratory virus illness syndromes and associated pathogens using historical data to identify diagnosis codes and other characteristics of healthcare visits most predictive of confirmed respiratory viral illnesses. The RAVIOLI algorithm comprises positive laboratory tests, evidence-based diagnosis codes, and measured fever. 

We have implemented RAVIOLI surveillance within the ESP automated public health surveillance platform to provide the Massachusetts Department of Public Health and participating practices with weekly reports on RAVIOLI incidence and contributing pathogens. RAVIOLI provides the department and practices with granular insight into evolving trends in respiratory viral illness rates that both retains the best features of traditional syndromic surveillance (capacity to monitor changes in disease incidence in near real time regardless of whether persons get tested) and simultaneously broadens the scope of surveillance to include multiple pathogens, not just influenza and SARS-CoV-2. The data provide insight into the relative proportions of contributing pathogens across multiple clinical facilities using both test results and diagnosis codes to identify organisms. 

When implemented well, syndromic surveillance provides a picture of the frequency, intensity, and trends in indicators of infectious and noninfectious conditions at local and extended scales. Integrating available viral pathogen test results, even if only in a subset of the population under surveillance, as we have done with the RAVIOLI algorithm, can add information about what is or is not contributing to observed increases in respiratory viral activity. Although influenza-like illness and COVID-like illness surveillance have been critical components for monitoring influenza and COVID-19 activity, reliance on fever as a required component of syndromic definitions is problematic because fever occurs only in a minority of laboratory-confirmed influenza and SARS-CoV-2 cases (*22*–*24*). Syndromic surveillance algorithms that require fever can therefore miss critical trends in the incidence of illnesses (*9*). The RAVIOLI algorithm, in contrast, does not require fever as a criterion and uses both laboratory test results and an evidence-based set of diagnosis codes to increase both sensitivity and specificity. 

Limitations of RAVIOLI surveillance include its development in a single region of the country using data from just 3 practice groups. Generalizability to other practice groups and regions need to be assessed. Changes in testing practices or coding practices over time and between practices might change the future performance of the RAVIOLI algorithm. The algorithm will require periodic revalidation and possibly modification. Furthermore, the breadth of pathogen capture using the RAVIOLI algorithm depends on the range and frequency of respiratory viral testing by clinicians; greater use of multiplex testing platforms will provide more granular and robust results. RAVIOLI surveillance is limited to patients who seek care, which likely biases the data toward pathogens associated with more severe disease. The PPV of algorithm components may vary by season; whether and how this affects surveillance should be considered. We used a 10% PPV threshold to select diagnosis codes for inclusion. This threshold was arbitrary, but we found using higher thresholds dramatically reduced the number of eligible diagnosis codes. We also found that the terms associated with diagnosis codes with a PPV of ≥10% were specific in their descriptions and not indicative of broad health conditions. However, the PPV threshold for including diagnosis codes should be considered in future revalidation of the algorithm. 

The healthcare site data included in developing the algorithm and whose data are part of the weekly reports came from both ambulatory and inpatient care facilities. We observed variation in which RAVIOLI categories (e.g., influenza, RSV) of the algorithm were detected at each site (data not shown). The limited number of sites makes it difficult to know if apparent differences between ambulatory and inpatient sites resulted from differences in catchment populations, illness severity associated with different viruses, or testing platforms. As the network expands to include a greater number and variety of sites, we plan to examine this question further. 

The Massachusetts Department of Public Health has used data from the underlying EHR-based system for infectious disease reporting and surveillance for more than a decade (*18*–*21*,*25*–*28*). This system has been sustained and enhanced over time to meet MDPH needs. As public health agencies consider what they need for the monitoring of current, emerging, and as-yet unidentified pathogens, we have found that a robust EHR data platform is a critical complement to traditional surveillance data. 

In conclusion, we developed an integrated, routine, automated EHR-based system for respiratory virus surveillance in Massachusetts. As experience with this approach expands, the hope is that this system will provide early indications of emerging infection trends and prevailing pathogens that render a fuller picture of respiratory viral activity beyond ILI and COVID-like illnesses. A broader view of circulating pathogens will provide public health agencies and healthcare institutions with more precise information useful for informing testing guidance, optimizing health communications; developing more targeted prevention activities, including vaccination; initiating enhanced infection control measures, such as masking and posting of notices in facilities; and generating other policies optimized to minimize the effect on population health of specific circulating pathogens. 

Appendix Additional information on development of an electronic health record–based algorithm for surveillance of respiratory virus–like illnesses.
