# Supplementary material for: Electronic Health Record–Based Algorithm for Monitoring Respiratory Virus–Like Illness
Source: Emerg Infect Dis. 2024 Jun;30(6):1096–103. doi: 10.3201/eid3006.230473 (PMC11138993; doi:10.3201/eid3006.230473)
Supplement: Appendix — Additional information on development of an electronic health record–based algorithm for surveillance of respiratory virus–like illnesses. [file 23-0473-Techapp-s1.pdf]

Article DOI: https://doi.org/10.3201/eid3006.230473

EID cannot ensure accessibility for Appendix materials supplied by authors. Readers who have difficulty accessing Appendix content should contact the authors for assistance.

Electronic Health Record–Based Algorithm for Respiratory Virus–Like Illness

Appendix

Appendix Table 1. ICD-10-CM diagnosis codes included in algorithm for influenza-like illness (ILI)

ICD-10 code	Description
Influenza-like illnesses	
B33.8	Other specified viral diseases
B34.1	Enterovirus infection, unspecified
B34.2	Coronavirus infection, unspecified
B34.4	Papovavirus infection, unspecified
B34.8	Other viral infections of unspecified site
B97.19	Other enterovirus as the cause of diseases classified elsewhere
B97.29	Other coronavirus as the cause of diseases classified elsewhere
B97.89	Other viral agents as the cause of diseases classified elsewhere
J00	Acute nasopharyngitis
J02.9	Acute pharyngitis, unspecified
J04.0	Acute laryngitis
J04.10	Acute tracheitis without obstruction
J04.11	Acute tracheitis with obstruction
J04.2	Acute laryngotracheitis
J05.0	Acute obstructive laryngitis
J06.0	Acute laryngopharyngitis
J06.9	Acute upper respiratory infection, unspecified
J20.9	Acute bronchitis, unspecified
J21.8	Acute bronchiolitis associated with other specified organisms
J21.9	Acute bronchiolitis, unspecified
J39.8	Other specified diseases of upper respiratory tract
J39.9	Disease of upper respiratory tract, unspecified
J12.89	Other viral pneumonia
J12.9	Viral pneumonia, unspecified
J13	Pneumonia associated with Streptococcus pneumoniae
J18.1	Lobar pneumonia, unspecified organism
J15.20	Pneumonia associated with staphylococcus, unspecified
J15.211	Pneumonia associated with methicillin susceptible staph
J15.212	Pneumonia associated with Methicillin resistant Staphylococcus aureus
J15.29	Pneumonia associated with other staphylococcus
J17	Pneumonia in diseases classified elsewhere
J18.0	Bronchopneumonia, unspecified organism
J18.8	Other pneumonia, unspecified organism
J18.9	Pneumonia, unspecified organism
J10.00	Influenza associated with other ident influenza virus with unspecified type of pneumonia
J10.08	Influenza associated with other ident influenza virus with other pneumonia
J11.00	Influenza associated with unidentified influenza virus with unspecified type of pneumonia
J11.08	Influenza associated with unidentified influenza virus with specified pneumonia
J12.9	Viral pneumonia, unspecified
J10.01	Influenza associated with other ident influenza virus with same other identified influenza virus pneumonia
J10.1	Influenza associated with other identified influenza virus with other respiratory manifestations
J11.1	Influenza associated with unidentified influenza virus with other respiratory manifestations
J10.2	Influenza associated with other identified influenza virus with GI manifestations
J10.81	Influenza associated with other identified influenza virus with encephalopathy
J10.82	Influenza associated with other identified influenza virus with myocarditis
J10.83	Influenza associated with other identified influenza virus with otitis media
J10.89	Influenza associated with other identified influenza virus with other manifestations
J11.2	Influenza associated with unidentified influenza virus with GI manifestations
J11.81	Influenza associated with unidentified influenza virus with encephalopathy

ICD-10 code	Description
J11.82	Influenza associated with unidentified influenza virus with myocarditis
J11.83	Influenza associated with unidentified influenza virus with otitis media
J11.89	Influenza associated with unidentified influenza virus with other manifestations
R07.0	Cough
R05	Pain in throat
Fever	
R50.2	Drug induced fever
R50.8	Other specified fever
R50.84	Febrile nonhemolytic transfusion reaction
R50.9	Fever, unspecified
R56.00	Simple febrile convulsions

Appendix Table 2. Patient counts and percentages meeting inclusion criteria for select RAVIOLI algorithm categories, January 2023–January 2024

	COVID-19			Influenza			Respiratory syncytial virus			Nonspecific	
Week	N	Positive lab test, %	Diagnosis code, %	N	Positive lab test, %	Diagnosis code, %	N	Positive lab test, %	Diagnosis code, %	N	Fever, %
Jan 1	4,128	50	74	786	71	65	62	77	32	1,011	22
Jan 8	3,550	49	75	532	73	69	42	79	38	1,031	27
Jan 15	2,680	51	75	289	66	75	28	79	39	847	26
Jan 22	2,647	47	78	278	73	78	35	83	37	973	29
Jan 29	2,425	47	76	257	70	81	25	76	32	953	31
Feb 5	2,446	47	78	225	73	81	25	76	44	1,029	31
Feb 12	2,235	45	80	194	71	77	22	73	50	1,025	32
Feb 19	1,841	38	83	129	78	73	14	86	29	850	32
Feb 26	1,863	37	83	145	74	83	23	70	61	1,019	33
Mar 5	1,649	37	83	136	76	82	20	80	35	1,040	33
Mar 12	1,546	35	84	134	84	75	11	91	18	954	34
Mar 19	1,594	35	84	165	80	76	7	86	43	1,016	33
Mar 26	1,531	30	88	149	85	74	7	86	29	1,074	37
Apr 2	1,499	30	89	153	80	77	11	55	73	1,034	36
Apr 9	1,350	28	88	110	83	72	9	56	67	1,038	39
Apr 16	1,122	23	90	106	75	77	12	75	33	802	38
Apr 23	1,124	29	87	104	81	74	7	57	43	854	35
Apr 30	1,189	29	89	94	81	78	2	0	100	833	36
May 7	1,107	26	90	91	85	73	5	60	40	901	42
May 14	1,121	25	90	88	82	75	4	75	50	847	34
May 21	1,168	25	91	100	80	79	1	0	100	873	37
May 28	1,044	27	91	60	80	73	3	100	33	820	39
Jun 4	1,176	28	90	72	83	82	5	60	40	803	39
Jun 11	1,052	29	90	68	72	81	3	67	33	748	44
Jun 18	938	24	91	59	78	78	1	100	100	694	44
Jun 25	814	24	90	65	86	72	6	67	67	691	41
Jul 2	757	30	88	46	74	78	3	33	67	534	43
Jul 9	908	30	87	47	89	77	2	100	0	564	48
Jul 16	919	35	87	43	79	79	2	100	0	591	47
Jul 23	1,035	38	85	45	82	78	10	100	30	638	47
Jul 30	1,163	38	84	58	86	76	9	89	44	560	44
Aug 6	1,396	44	83	38	74	84	5	80	40	579	43
Aug 13	1,474	45	83	49	88	78	5	80	60	591	36
Aug 20	1,730	47	82	62	84	84	17	100	18	585	34
Aug 27	1,921	47	82	61	85	84	12	92	25	575	34
Sep 3	1,837	47	82	51	84	75	17	82	47	609	33
Sep 10	2,117	44	82	81	78	80	25	96	28	717	30
Sep 17	2,303	42	82	97	78	84	30	90	43	837	31
Sep 24	2,372	39	86	87	80	83	39	92	28	859	23
Oct 1	2,326	39	85	100	77	78	55	95	31	955	26
Oct 8	2,123	32	87	101	76	83	72	85	38	1,019	27
Oct 15	2,038	30	88	113	74	81	101	89	35	1,055	24
Oct 22	2,020	27	90	125	78	74	162	92	36	1,080	24
Oct 29	1,863	24	91	131	73	82	196	85	35	1,130	23
Nov 5	1,945	23	91	174	88	72	278	87	38	1,226	25
Nov 12	2,096	24	92	179	80	72	290	87	37	1,268	24
Nov 19	1,927	25	90	191	85	69	258	83	45	958	24
Nov 26	2,717	29	87	371	82	73	318	85	40	1,294	21

	COVID-19			Influenza			Respiratory syncytial virus			Nonspecific	
Week	N	Positive lab test, %	Diagnosis code, %	N	Positive lab test, %	Diagnosis code, %	N	Positive lab test, %	Diagnosis code, %	N	Fever, %
Dec 3	2,875	32	88	573	87	64	313	88	32	1,249	26
Dec 10	3,245	37	86	806	85	68	269	86	35	1,270	26
Dec 17	3,685	40	85	1,043	85	67	203	83	32	1,189	26
Dec 24	3,348	45	82	938	87	66	165	89	24	939	25
Dec 31	3,551	39	83	880	81	70	131	83	32	909	26
Jan 7 (2024)	3,202	40	83	847	80	73	104	87	28	956	27

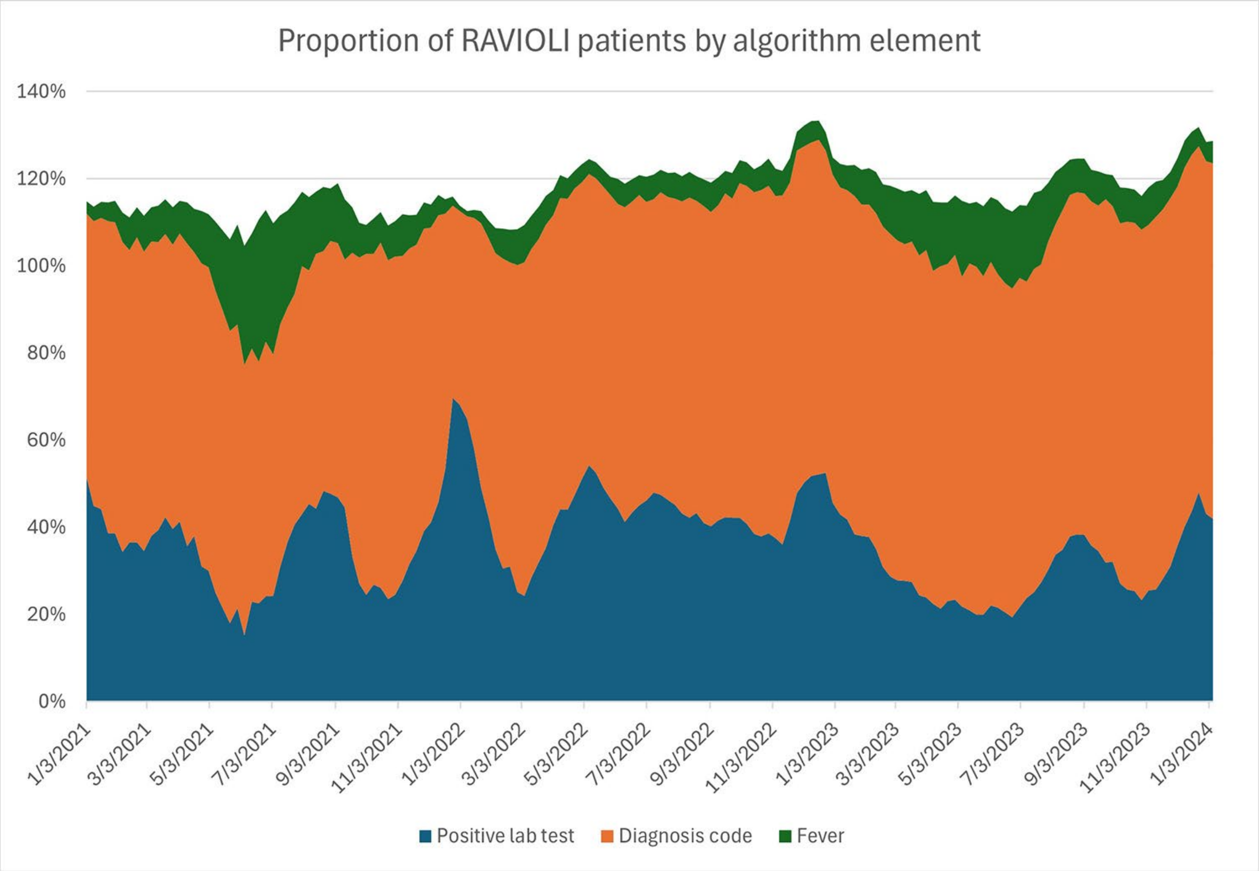

Appendix Figure. Proportion of RAVIOLI patients by algorithm element, January 2021–January 2024. Data include all categories of RAVIOLI and show the proportion identified on the basis of a positive laboratory test, diagnosis code, and/or fever. Patients could meet >1 criterion (e.g., have both a positive laboratory test and a diagnosis code).
